# A Novel Polymeric Nanohybrid Antimicrobial Engineered by Antimicrobial Peptide MccJ25 and Chitosan Nanoparticles Exerts Strong Antibacterial and Anti-Inflammatory Activities

**DOI:** 10.3389/fimmu.2021.811381

**Published:** 2022-01-19

**Authors:** Yu Haitao, Chen Yifan, Sun Mingchao, Han Shuaijuan

**Affiliations:** ^1^ Institute of Systems Biomedicine, Department of Immunology, School of Basic Medical Sciences, Beijing Key Laboratory of Tumor Systems Biology, Peking University Health Science Center, Beijing, China; ^2^ College of Animal Science and Technology, Hebei Agricultural University, Baoding, China; ^3^ State Key Laboratory of Animal Nutrition, Institute of Animal Sciences of Chinese Academy of Agricultural Sciences, Beijing, China

**Keywords:** antibiotic-resistant microorganisms, antimicrobial polymeric nanoparticles, chitosan nanoparticles-MccJ25, anti-inflammatory activity, antibacterial activity, tetracycline-resistant enterotoxigenic *E. coli*, lipopolysaccharides, RAW264.7 macrophages

## Abstract

Infection caused by antibiotic-resistant microorganisms (ARMs) has been declared a global threat to public health. Polymeric nanoparticles (PNPs) formed by antimicrobial peptides (AMPs) and synthetic PNPs against ARM infections are emerging. PNPs are also considered to be a promising natural biological preservative that prevents microbial spoilage through food processing and preservation. We engineered CNMs, a novel nanocomposite antibacterial agent based on chitosan nanoparticles and AMP microcin J25. In this study, we aimed to evaluate the comprehensive antimicrobial activity, potential antimicrobial mechanism, and anti-inflammatory activity of CNMs. We demonstrated that CNMs harbor excellent bactericidal activity against clinical foodborne pathogens and ARMs. CNMs caused fast mortality against different growth phases of tetracycline (Tet)-resistant enterotoxigenic *E. coli* (ETEC) and significantly killed Tet-resistant ETEC in food biological environments. Mechanistically, CNMs have the ability to bind lipopolysaccharides (LPS), neutralize endotoxin, and promote diaphragm permeability by damaging the cell membrane. CNMs did not cause mouse RAW264.7 cell cytotoxicity. Notably, CNMs significantly reduced the cytotoxicity of RAW264.7 macrophages induced by LPS. The LPS-induced inflammatory response was significantly ameliorated by CNMs by reducing the levels of nitric oxide and proinflammatory cytokines, including tumor necrosis factor α, interleukin (IL)-6, IL-8, IL-1β, Toll-like receptor 4, and nuclear factor κB (NF-κB), in LPS-challenged RAW264.7 macrophages. CNMs downregulated the NF-κB and mitogen-activated protein kinase signaling pathways, thereby inhibiting inflammatory responses upon LPS stimulation. Taken together, CNMs could be applied as effective antimicrobial/anti-inflammatory agents with lower cytotoxicity in food, medicine, and agriculture to prevent bacterial contamination and infection, respectively.

## Introduction

The increasing prevalence of bacterial drug resistance and the intensification of multi/super drug-resistant bacteria are among the largest public health problems in the world (1–4). Developed countries, such as the European Union, Japan, South Korea, the United States, and even developing countries, such as Thailand, have successively banned the use of antibiotics. The Chinese government officially announced the implementation of the ban on drug feed additives, indicating that China has entered the post-antibiotic era. Antibiotics play critical roles in reducing pathogenic microbial infection (5, 6). Either in the pre-antibiotic era or in the post-antibiotic era, bacterial infection is becoming the biggest killer (7). It is estimated that if effective action is not taken, 10 million people will die of bacterial drug resistance by 2050, and the cumulative global economic cost may reach US $100 trillion (8, 9). Additionally, this era means that the infection of pathogenic microorganisms has a serious impact on the food, water, feed, and environment. Animals and humans are easily infected and circulate by pathogenic bacteria (7, 10–12).

An increasing problem of drug resistance facilitates the development of effective, safe, and novel-acting alternatives to cure microbacillary infections with grievous antibiotic resistance (11–13). In the post-antibiotic era, emerging nanobiomaterials as antimicrobials have exhibited potential for the treatment of severe drug-resistant bacterial infections due to their excellent properties, such as size effects, special physical and chemical properties, and easy modification. For instance, antimicrobial peptides (AMPs) and antimicrobial polymers provide a new way to solve drug-resistant microorganisms, which has important medical significance in the case of increasingly serious drug resistance (14–18). They have strong antibacterial activity and stability and lower toxicity. Polymer nanoparticles (PNPs), formed by AMPs and nanoparticles, have a high profile because of their effective antibacterial capacity and do not induce bacterial mutations or drug resistance. They can be directly used as antibacterial agents and are considered promising antibiotic substitutes (19–21).

Microcins, a class of bacteriocins, and chitosan nanoparticles (CNs), as potential clinical antibiotics and immunomodulators, have attracted increasing attention (22–31). In previous studies, we successfully designed and engineered new PNPs based on AMP MccJ25 and CNs (CNMs). We proved that engineered CNMs have strong bactericidal activity against a variety of microorganisms, including clinically significant *Escherichia coli* (*E. coli*) K88, *E. coli* O157, and methicillin-resistant *Staphylococcus aureus*, with low toxicity to mammalian cells and *Caenorhabditis elegans* (32). Critically, CNMs exert strong antibacterial activity without raising resistant mutants and do not increase resistance. However, detailed scientific information regarding the mode of action and antimicrobial and anti-inflammatory properties has not been sufficiently understood. In particular, the potential applications of CNMs against ARMs remain unclear. To address this issue, in this study, we further studied the comprehensive antimicrobial activity and potential anti-inflammatory activity of CNMs. These findings will provide the potential application of PNPs, a novel type of therapeutic molecule, in the real world in the areas of food, health, and agriculture for treating infectious and inflammatory diseases caused by ARMs.

## Materials and Methods

### Preparation of CNs-MccJ25 Conjugates (CNMs)

Chitosan nanoparticles were synthesized as previously developed methods with fewer corrections (27). In short, 2% chitosan (w/v; 448869, Sigma-Aldrich, St. Louis, MO) was resolved with 0.1 M acetic acid (2%, v/v; Thermo Fisher Scientific Inc., Waltham, MA). Then, 1% Tween 80 (v/v; Acros Organics, Morris, NJ) was added to the above compounds. After the chitosan was completely dissolved, the chitosan solution was sonicated with 10% sodium sulfate (w/v; Thermo Fisher Scientific Inc.) for cross-linking. The acoustic degradation continued for 25 min. Ultimately, the sonicated solution was centrifuged at 14,000 rpm for 10 min to obtain CNs. CNs were resuspended three times with aseptic ddH2O and accommodated at 4°C before conjugation with AMP microcin J25 (MccJ25) following a previous protocol described previously (32). AMP MccJ25 was provided by our previous research team, Professor Qiao lab. MccJ25 contain 21 amino acids; the weight and sequence of MccJ25 were 2107 Da and GGAGHVPEYFVGIGTPISFYG, respectively.

Conjugation of MccJ25 to CNs is as follows: 0.1 M sodium acetate (Thermo Fisher Scientific Inc.) buffer was applied to decompose CNs. According to the CN : MccJ25 ratio = 10:1, a MccJ25 solution was added to the above solution (w/w). 1-Ethyl-3-(3-dimethylaminopropyl)carbodiimide (EDC; 0.5 mM; Sigma-Aldrich, St. Louis, MO) and sulfo-N-hydroxysulfosuccinimide (sulfo-NHS; 0.25 mM; Sigma-Aldrich, St. Louis, MO) as a cross-linker were directly added into the mixture and churned at 4°C overnight. The conjugator CNMs were dialyzed using ddH2O (dialysis membrane molecular weight cutoff 12≥14 kDa). ddH2O was replaced by commensurable capacity every 2 h to 48 h. The dialyzed CNMs were lyophilized overnight to obtain lyophilized powdered CNMs, dissolved in sterile Milli-Q water, and maintained at 4°C until the end of the test.

### Minimum Inhibitory Concentration Test

The minimum inhibitory concentration (MIC) of CNMs against pathogenic bacteria was measured using the broth microdilution method in compliance with clinical and Laboratory Standards Institute (CLSI) guidelines (33). CNMs were inoculated into 200 μl of Mueller Hinton broth (MHB; Difco™, BD & Co., East Rutherford, NJ) to obtain final concentrations of 0%, 0.0125%, 0.025%, 0.05%, 0.1%, and 0.2%. Tetracycline-resistant enterotoxigenic *E. coli* (Tet-resistant ETEC), *E. coli* K88, *Salmonella enteritidis*, *Salmonella pullorum*, *Salmonella typhimurium*, and *E. coli* O157 were used as the indicated pathogenic bacteria in MIC analysis. All bacteria were cultured to logarithmic phase and inoculated into medium with different concentrations of CNMs, and the final concentration of bacteria was 5 × 10^5^ cfu/ml. Ninety-six-well microtiter plates were cultured at 37°C and shaken at 200 rpm overnight. MIC was tested as the lowest concentration of CNMs that inhibited bacterial growth. The experiment was repeated three times.

### Time-Killing Curves of CNMs Assay

The killing kinetics of bacteria, including Tet-resistant ETEC, *E. coli* O157, *E. coli* K88, *Salmonella enteritidis*, *Salmonella pullorum*, and *Salmonella typhimurium*, were determined as previously described (25). Briefly, log-phase bacterial cells above a single colony were diluted in fresh Luria–Bertani (LB) medium to a final concentration of 5 × 10^4^ cfu/ml. Bacterial cells were cultured with different concentrations of CNMs (1, 2, or 4 × MIC) for 0, 2, 4, 6, and 8 h, and then 0.1 ml of bacterial cultures was serially diluted, plated on an LB plate, and cultured at 37°C overnight. The bacterial colonies were counted. Three repetitions were performed in three independent experiments.

### Live/Dead Assay

The live/dead test was performed according to the protocol of the LIVE/DEAD BacLight Bacterial Viability Kit 7 (Molecular Probe company, Eugene) to detect bacterial viability. In short, 5 × 10^5^ cfu/ml Tet-resistant ETEC was transferred into 1 ml of fresh LB containing 1 × MIC CNMs. After that, the bacterial culture was cultured at 37°C for 2 h and then cultured in the dark with SYTO 9 and propidium iodide at room temperature for 15 min. Bacterial viability was observed using a fluorescence microscope (EVOS XL Cell Imaging System).

### Activity of CNMs to Different Growth Phases of Tet-Resistant ETEC

Single colonies resistant to Tet ETEC were transferred to 5 ml LB and incubated overnight at 200 rpm at 37°C. The next day, the culture was diluted to fresh LB at 1:100 and immediately cultured at 37°C. To test the antibacterial activity of CNMs on Tet-resistant ETEC at different growth stages, bacteria were cultured until they reached the early logarithmic (OD600 = 0.5), late logarithmic (OD600 = 1.0), or stationary (OD600 = 3.0) stage. Approximately 10^8^ cfu/ml Tet-resistant ETEC at different stages was inoculated into different concentrations of CNMs (0, 1, 2, and 4 × or 8 × MIC for 0, 2, 4, 6, 12, and 24 h). After culture, the cultures were continuously diluted with phosphate-buffered saline (PBS), inoculated on LB agar, and cultured overnight at 37°C to count cfu. Two repeated tests were conducted in three independent tests.

### Antimicrobial Activity of CNMs in Different Conditions

The anti-Tet-resistant ETEC effects of CNMs on biological foods, including sterile skim milk (SKM), egg yolk (SEY), and minced meat (pork) extract (SMS), were evaluated as described previously, with slight modifications (25). Tet-resistant ETEC at the stationary phase was inoculated into biological product samples to a final concentration of 10^6^ cfu/ml, and then CNMs were mixed with a final concentration of 8 × MIC mixtures for comparison. The control group was treated with sterile normal saline (SSW) without CNMs. After shaking at 37°C (200 rpm) for 2 h, the active cells were tested. After culture, Tet-resistant ETEC was collected and continuously diluted with sterile PBS, and bacterial liquid was inoculated on the culture medium. LB agar culture dishes were kept at 37°C overnight to count the number of colony. Colonies were counted to determine cfu/ml. The experiment was repeated in three independent experiments.

In synthetic gastrointestinal fluids, including simulated gastric fluid (SGF) and simulated intestinal fluid (SIF) were individually prepared as described in the Chinese Pharmacopoeia (25). SGF consisted of 10 mg/ml pepsin in 0.03 M NaCl with pH values of 1.5 and 2, respectively, whereas SIF consisted of 10 mg/ml pancreatin in 0.05 M KH_2_PO4 with a pH of 7.5. A final concentration of 5 × 10^8^ cfu/ml of stationary-phase Tet-resistant ETEC was inoculated initially, and the log reduction was assessed after 1 h incubation at three different final CNMs concentrations (0.025%, 0.05%, and 0.1%).

### Antimicrobial Mechanisms of CNMs

#### LPS-Binding Affinity

The lipopolysaccharide (LPS)-binding activity of CNMs was tested by the BODIPY-TR cadaverine (BC, Sigma, USA) replacement test, as described earlier and with slight modifications. *E. coli* O111: B4 LPS (Sigma, USA) at a final concentration of 25 µg/ml and BC dye at a final concentration of 2.5 µg/ml were cultured in Tris buffer (pH 7.4) for 5 h. The same amounts of different concentrations of CNMs and LPS-BC dye medium were mixed in 96-well black plates and then cultured at 37°C for 70 min. Excitation and emission at λ = 620 nm and λ = 580 nm, respectively, were used to monitor the fluorescence using a fluorescence spectrophotometer (Infinite 200 PRO, Tecan, China). The experiment was carried out independently three times and repeated three times each time. The LPS-binding affinity of CNMs was converted to % Δ F (AU) as follows:


$$\%\;\Delta F\;\left(AU\right)\;=\;\left(F_{obs}-\;F_{0}\right)/\left(F_{100}-F_{0}\right)\times 100$$

where F_obs_ is the BC fluorescence indicating the concentration of CNMs obtained, F_0_ is the BC initial fluorescence of *E. coli* O111:B4 LPS without CNMs, and F_100_ is 10% added after using 10 μg/ml polymyxin B (Sigma, USA) as the positive control BC fluorescence.

#### Measurement of LPS Neutralization

CNMs neutralizing endotoxin were evaluated by chromogenic limulus lysate (LAL) analysis according to the manufacturer’s instructions, with some modifications. Constant endotoxin (1 EU/ml) was cultured in different media. Different concentrations of CNMs (0%, 0.025%, 0.05%, 0.1%, 0.2%, and 0.4%) in the wells of pyrogen sterile microtiter plates were heated at 37°C. Then, 50-µl aliquots of concentrated LAL reagent were added and incubated at 37°C for 10 min. The 100-µl color matrix was yellow. The reaction was stopped by adding 25% hydrochloric acid, and the absorbance was measured at 545 nm.

#### Outer Membrane Permeabilization Assay

According to the established protocol form the kit’s description, the outer membrane permeability of CNMs on Tet-resistant ETEC of stationary phase was determined using N-phenyl-1-naphthylamine (NPN) dye. Tet-resistant ETEC of the log phase was obtained by centrifugation and washed 3 times with HEPES buffer containing 5 mM glucose (pH 7.4). Bacterial cells were resuspended in the same buffer at a final concentration of 10^6^ cfu/ml and treated with NPN dye at a final concentration of 10 μM, and the bacterial cells were stained. In 96-well black plates, bacterial suspensions were treated with CNMs at 0%, 0.025%, 0.05%, 0.1%, and 0.2%. A fluorescence spectrophotometer (Infinite 200 PRO, Tecan, China) at excitation λ = 422 nm and emission λ = 350 nm was applied to record fluorescence. The data are converted to the NPN absorption percentage as follows:


$$\%\;NPN\;uptake\;=\;\left(F_{obs}-\;F_{0}\right)/\left(F_{100}-F_{0}\right)\times 100$$

where F_obs_ is the fluorescence obtained at the concentration indicating CNMs, F_0_ is the initial fluorescence of NPN in Tet-resistant ETEC cells without CNMs, F_100_ is added after 10 μg/ml polymyxin B (Sigma, USA) NPN fluorescence, and polymyxin B was used as a positive control to cause outer membrane permeability.

#### Transmission Electron Microscope Analysis

A final concentration of 10^6^ cfu/ml Stationary-phase Tet-resistant ETEC cells was initially prepared. Bacterial cells were treated with 0.025%, 0.05%, and 0.1% CNMs for 1 h at 37°C. Sterile PBS without CNMs was used as a control treatment. Bacterial cells were washed with 2.5% (v/v) glutaraldehyde at 4°C and fixed overnight. The next day, the cells were dehydrated and fixed with a graded ethanol series (50%, 70%, 90%, and 100%) for 10 min, and the bacterial cells were washed twice with PBS and fixed with 2% osmium tetroxide and PBS for 80 min. The samples were dehydrated in a graded ethanol series (50%, 70%, 90%, and 100%) for 8 min and then treated with 100% ethanol, a mixture of 100% ethanol and acetone (1:1, v/v), and absolute acetone for 10 min. The sample was added to a 1:1 (v/v) mixture of acetone and epoxy resin for 30 min and then soaked in pure epoxy resin overnight. It was sliced with an ultrathin slicer, stained with uranyl acetate and lead citrate, and observed by transmission electron microscopy (TEM, Hitachi H-7650, Japan).

#### DPPH Free Radical Scavenging Assay of CNMS

Twenty-four milligrams of 2,2-diphenyl-1-picrylhydrazyl (DPPH, GlpBio Technology, Montclair, CA, USA) powder was prepared and dissolved in 100 ml absolute ethanol. Vitamin C (VC, GlpBio Technology, Montclair, CA, USA) and butylated hydroxytoluene (BHT, GlpBio Technology, Montclair, CA, USA) were used as positive controls. Equal columns of CNMs at desired concentrations were transferred to the DPPH solution in the tested tube (A_i_). The mixtures were shaken well and put in the dark at room temperature for 30 min. Equal columns of CNM solution were used as a control (A_j_). Equal columns of ethanol were incubated with DPPH solution as a blank (A_0_), and then absorbance was determined using a Q-6 UV–Visable Spectrophotometer at 517 nm (Shanghai Metash Instruments Co., Ltd., Shanghai, China). Data were calculated as the following equation:


$$\text{DPPH scavenging rate}=(1-\left({\text{A}}_{\text{i}}-{\text{A}}_{\text{j}}\right)/{\text{A}}_{0}\times \;100$$

### Evaluation of Cytotoxicity of CNMs to RAW264.7 Cells

#### Cell and Culture Conditions

Mouse RAW264.7 cells were cultured in Dulbecco’s modified Eagle’s medium (DMEM; Corning, Corning, New York) with 10% fetal bovine serum (FBS; Clone, Logan, UT). Cells were cultured at 37°C and 5% CO_2_ until 80%–90% fusion. Before CNM treatment, cells were inoculated in 24-well or 96-well plates for 24 h.

#### Cell Morphology Analysis

To test whether CNMs affect host health, RAW264.7 cell lines were assessed for toxicity risk. Cells were treated with 0.025%, 0.05%, and 0.1% CNMs, incubated for 12 h, and then treated with Dulbecco’s phosphate-buffered saline (DPBS; HyClone, Logan, UT). 1% Triton X-100 (Sigma-Aldrich, St. Louis, MO) was used as a positive control. Images of cell morphology were taken by an EVOS XL cell imaging system. (Thermo Fisher Scientific Inc.,Waltham, MA)

#### Cell Viability Analysis

To assay whether CNMs caused membrane damage in RAW264.7 cells, an LDH Cytotoxicity Detection Kit (Clontech, Mountain View, CA) was applied to determine lactate dehydrogenase (LDH) release. In short, 2 × 10^4^ RAW264.7 cells were inoculated in 96-well plates until 80%–90% confluence. Various concentrations of CNMs (0.5 ×, 1 ×, 2 ×, 4 × and 8× MIC) were added to the cells, and 1% Triton X-100 was used as a positive control. 100 μl was removed from each well cell culture medium and transferred to the new well. The catalyst and dye solution were added to the medium and incubated for 30 min. A microplate reader (Synergy Mx, BioTek, Winooski, VT) was applied to measure the absorbance at 490 and 670 nm. The absorbance value was corrected by subtracting the reading at 670 nm from the reading at 490 nm. Absorbance values were standardized by 1% Triton X-100 treatment (100% cell death) and negative control (0% cell death).

#### MTT Assay

An MTT cytotoxicity test kit (Eugene Life Technology, Oregon, USA) was used to detect whether CNMs affect cell mitochondrial metabolic activity. A total of 10^4^ cells were added to 96-well plates and grown to an 80%–90% fusion rate within 24 h. Then, CNMs at the same concentration as that used in LDH analysis were added to the cells and cultured for 24 h. 1% Triton X-100 was used as positive control. Then, 20 μl of MTT solution (5 mg/ml in DPBS) was added to each well and incubated at 37°C for 3.5 h with 5% carbon dioxide. MTT metabolites were collected by centrifugation and then suspended in 200 μl dimethyl sulfoxide. The optical density was read at 560 nm, and the background was subtracted at 670 nm. Absorbance values were standardized by 1% Triton X-100 treatment (100% cell death) and negative control (0% cell death).

#### Assessment of Cytotoxicity Effects of CNMs on LPS-challenged RAW264.7 Cells

Mouse RAW264.7 macrophages were grown under 80%–90% confluence, then cells were diluted using DMEM medium to 10^4^ cells/ml and transferred, and the 24-wells were cultured overnight. After culture, CNMs at different concentrations were added to the medium and cultured for 24 h, then 1 µg/ml LPS was applied to treat cells and cultured for 8 h. Untreated cells in the absence of LPS and CNMs were used as control. LPS-treated cells (1 × 10^4^ cells/ml) in the presence and in the absence of different concentrations of CNMs was used as challenged treatment. Then, LDH and MTT assays were applied to evaluate the anti-LPS-induced cytotoxicity of CNMs.

#### CNMs Inhibited Nitric Oxide Secretion in LPS-Induced RAW264.7 Cells

RAW264.7 macrophages treated with 1 µg/ml LPS were incubated with CNMs (0.0125%, 0.025%, and 0.05%) at different concentrations. Culture supernatant was collected at 24 h after culture, and the contents of nitric oxide (NO) and tumor necrosis factor-α (TNF-α), interleukin (IL)-6, and IL-1β were detected.

#### Quantitative Real-Time PCR

Proinflammatory cytokines, including TNF-α, IL-6, IL-8, IL-1β, Toll-like receptor 4 (TLR4), and nuclear transcription factor-κB (NF-κB), in cells were detected by quantitative real-time PCR (qRT-PCR). The primers for the above genes were synthesized by Beijing Sanbo Zhiyuan Biotechnology Co., Ltd., and are provided in **Table S1**. The total RNA of cells was extracted completely according to the TRIzol kit and CryoGrinder. The concentration and purity of RNA were detected using a NanoDrop 2000. Then, the high concentration was diluted, repacked, and frozen in an appropriate proportion. Reverse transcription was conducted. This step is operated on ice all the way. RNA (1 μg) reverse transcription was performed according to the PrimeScript RT Reagent Kit with the gDNA Eraser Kit, resulting in a total volume of 200 μl cDNA. Then, PCR detection was performed, and TNF-α, IL-6, TLR4, and NF-κB were determined by a StepOnePlus real-time fluorescence quantitative PCR system (Takara, SYBR Premix DimerEraser, Perfect Real Time). β-Actin was used as a homekeeping reference. The relative mRNA expression of the target gene was determined by the 2^-ΔΔCT^ method, and the specific formula was ΔCT = CT (target gene) - CT (β-actin); ΔΔCT = Δ CT (test group) - △CT (control group); 2^-ΔΔCT^ = relative mRNA expression.

#### Western Blot

RIPA lysis buffer containing protease inhibitors (Applygen, Beijing, China) was applied to homogenize the treated and untreated cells. A BCA Protein Assay Kit (Thermo Fisher Scientific, Rockford, IL) was used to measure the protein concentrations of the samples. Approximately 30 μg of sample protein was electrophoresed on SDS-PAGE and electrotransferred onto PVDF membranes (Millipore). Membranes were blocked using 1 × TBST with 5% bovine serum albumin (Sigma-Aldrich, St Louis, MO) for 30 min at room temperature. Afterward, samples were incubated with the corresponding primary antibodies (1:5,000 dilutions overnight at 4°C) for TNF-α, phosphorylated p65, and phosphorylated p38 (Cell Signaling Technology, Boston, MA). TBST (1×) was used to wash the membrane 5 times, and the membranes were cultured with horseradish peroxidase-conjugated goat anti-mouse IgG (Huaxingbio Biotechnology, Beijing, China) for 15 min at room temperature. Chemifluorescence was detected with Western Blot Luminance Reagent (Applygen, Beijing, China) by an ImageQuant LAS 4000 mini system (GE Healthcare Biosciences AB, Inc., Sweden) and quantified by a gel imaging system with Image Quant TL (GE Healthcare Life Sciences).

### Statistical Analysis

These results are indicated as the mean ± standard error of the mean (SEM). Data were analyzed by one-way ANOVA with Prism 8 software (GraphPad Software Inc., San Diego, CA, USA). Tukey’s *post-hoc* test was applied to determine differences among treatments. *p* values < 0.05 indicated statistical significance. Unless otherwise specified, all experiments were carried out three independent times unless otherwise stated.

## Results

### CNMs Exert Strong Bactericidal Activities Against Pathogenic Bacteria

**Figure 1:**
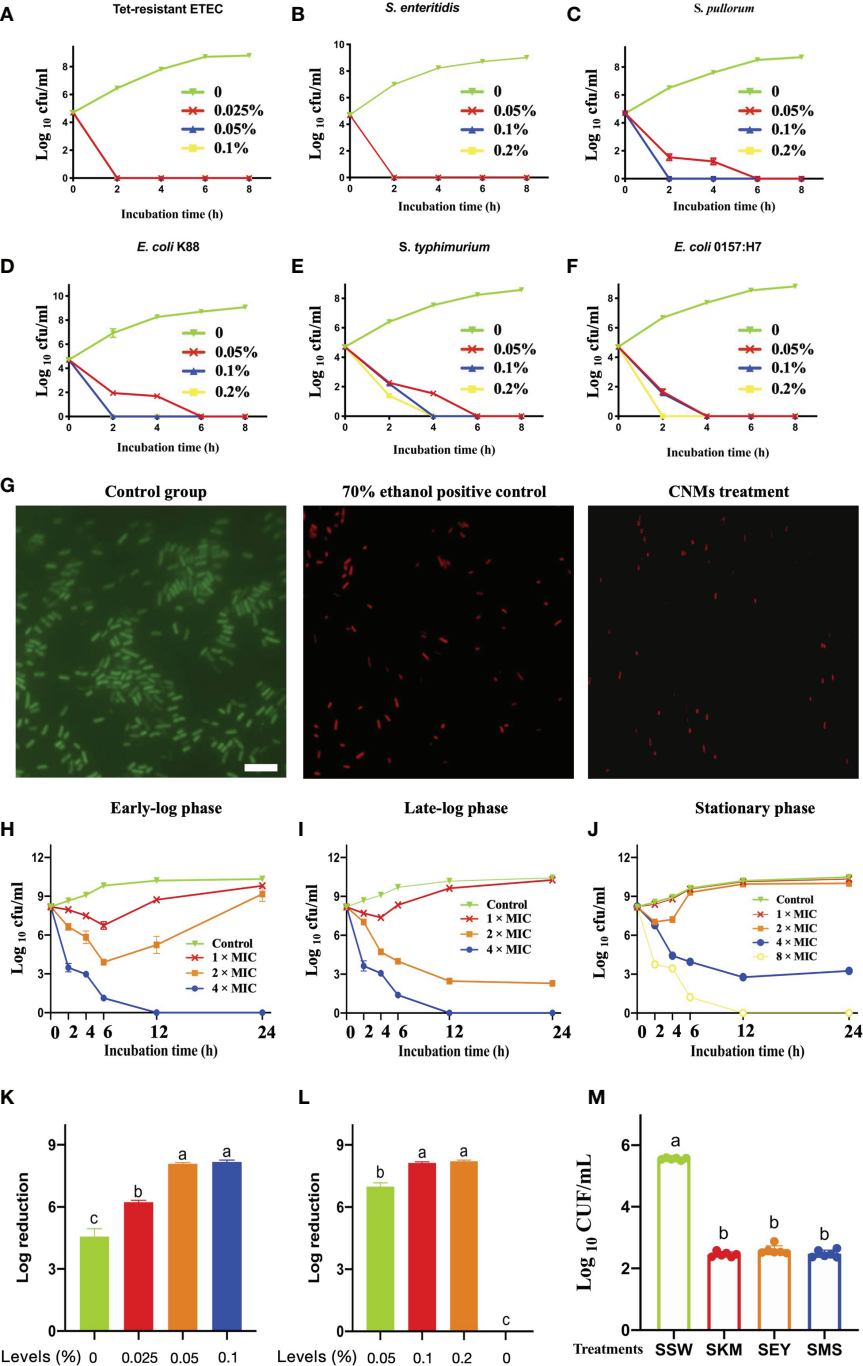
Comprehensive evaluation of antimicrobial activity of CNMs. Different indicated bacterials at approximately 5 × 10^4^ cfu/ml were cultured with different indicated concentrations of CNMs for various durations. After incubation, the concentration of bacteria was measured by counting the number of colonies. **(A–F)** Time-killing kinetics of different concentrations of CNMs toward pathogenic bacteria. **(A)** Tet-resistant ETEC: tetracycline-resistant enterotoxigenic *Escherichia coli.* **(B)** *S. enteritidis: Salmonella enteritidis*, **(C)** *S. pullorum: Salmonella pullorum*, **(D)** *Escherichia coli* K88, **(E)** *S. typhimurium: Salmonella typhimurium*, and **(F)** *Escherichia coli* O157. Data are presented three independent experiments. **(G)** The engineered CNMs kill tetracycline-resistant ETEC. Live/dead viability analysis of tetracycline-resistant ETEC challenged. Fluorescent micrographs of cells treated with 0% CNMs are shown on the left, 70% ethanol is shown in the middle, or CNM at the 1 × MIC level is shown on the right. The white bar indicates 6 µm. **(H–J)** Bactericidal activity of CNMs against Tet-resistant ETEC. The indicated bacteria were incubated in early-log **(B)**, late-log **(C)**,and stationary **(D)** phases. Approximately 5 × 10^8^ cfu/ml Tet-resistant ETEC cells were cultured with CNMs at various concentrations. Data are presented three independent experiments. **(K–M)** Bactericidal activity of CNMs at different concentrations in synthetic gastrointestinal fluids and food biological products. Approximately 5 × 10^8^ cfu/ml Tet-resistant ETEC was inoculated into fluids containing CNMs. **(K)** Log reduction of bacteria after 1 h in SGF (pH = 2). **(L)** Log reduction of bacteria in SIF-containing CNMs. **(M)** Antibacterial activity of CNMs against Tet-resistant ETEC in food biological products. Approximately 5 × 10^6^ cfu/ml of the indicated bacteria was inoculated into autoclaved skim milk (SKM), mincemeat supernatant (SMS), and sterilized egg yolk (SEY). Bacteria were treated after 2 h in bioenvironments containing 8 × MIC CNMs. Viable cells were counted. Data are indicated as the mean cfu/ml ± standard error for three individual experiments. Means with different lowercase letters differ (*p* < 0.05).

Based on the MIC value of CNMs against the indicated pathogens (**Table S2**), we aimed to further test the antibacterial activity of CNMs against different intestinal pathogens, and we conducted a time-course bactericidal assay (**Figures 1A–F**). Killing curves of CNMs toward pathogenic bacteria, including Tet-resistant ETEC (**Figure 1A**), *S. enteritidis* (**Figure 1B**), *S. pullorum* (**Figure 1C**), *E. coli* K88 (**Figure 1D**), *S. typhimurium* (**Figure 1E**), and *E. coli* O157 (**Figure 1F**), were evaluated. These indicated that pathogenic microorganisms were collected every 2 h during incubation to count viable cell numbers for 8 h. The results demonstrated that the bactericidal capacity of CNMs showed dose-dependent effects. CNMs at 2 × MIC completely killed *S. pullorum* and *E. coli* K88 after 2 h of incubation. However, for the pathogenic bacteria *S. typhimurium* and *E. coli* O157, CNMs killed them after 4 h of incubation at a 2 × MIC concentration. When the level of CNMs was lowered to 1 × MIC, CNMs completely eliminated Tet-resistant ETEC and *S. enteritidis* after 2 h of incubation. Overall, CNMs killed all bacteria after 2 or 4 h, and pathogenic bacteria did not present regrowth after 8 h of incubation. Consequently, the killing curve results have shown that CNMs exhibit excellent antimicrobial activity against intestinal pathogens.

According to the results of the time kill curve, a live/dead bacteriostasis test was carried out for further study. CNMs at 1 × MIC had bactericidal activity against Tet-resistant ETEC, and their potential application value in the treatment of intestinal infectious diseases caused by drug-resistant microorganisms was determined. As shown in **Figure 1G**, in the control group, the bacteria stained with SYTO 9 emitted green fluorescence, indicating that the bacteria were alive. Compared with the control group, the bacteria in the 70% ethanol and CNMs treatment groups were stained with propidium iodide and showed red fluorescence, suggesting that CNMs killed bacteria by damaging cell membranes because this dye can penetrate into the cytosol. CNMs showed excellent bactericidal activity against Tet-resistant ETEC tested by disrupting the cell membrane, providing promising perceptiveness as traditional antibiotic alternatives.

As shown in **Figure 1G**, CNMs damaged the Tet-resistant ETEC membrane. Therefore, we hypothesized that the bactericidal activity of CNMs is not related to the synthesis of the bacterial cell wall but directly destroys the bacterial cell wall. In addition, when foodborne bacterial pathogens enter host cells, the bacterial population is usually low (<10^6^/g source), but the pathogen will replicate to a higher number in its niche and thus exhibit infectious disease (30). To evaluate whether CNMs can also be used to treat disease in the late stage of infection, we simulated the late stage of infection with a large number of bacteria (5 × 10^8^ cfu/ml).

Subsequently, the bactericidal activity of CNMs toward Tet-resistant ETEC cells at different growth phases was employed to further test this hypothesis. The antibacterial activity of different concentrations of CNMs against Tet-resistant ETEC was tested at 2, 4, 6, 12, and 24 h at different growth phases, such as the early-log phase (**Figure 1H**), late-log phase (**Figure 1I**), and stationary phase (**Figure 1J**). The results revealed that CNMs showed significant bactericidal activity against Tet-resistant ETEC regardless of the bacterial growth phase. Tet-resistant ETEC cells at early log and late log were effectively killed by CNMs at 4 × MIC, Tet-resistant ETEC cells at early or late log phase were reduced to undetectable levels within 12 h when treated with 0.1% CNMs, and bacterial cells did not present regrowth after 48 h of treatment. Compared to late-log phase Tet-resistant ETEC, CNMs of 2 × MIC also effectively exhibited bactericidal activity. However, stationary-phase Tet-resistant ETEC cells showed significantly greater resistance to CNMs than early- and late-log-phase Tet-resistant ETEC cells, and CNMs killed the stationary-phase Tet-resistant ETEC cells to non-detectable levels by 12 h at an 8 × MIC concentration. When the late-log and stationary-phase cells of Tet-resistant ETEC incubated with 2 × MIC CNMs could not be killed completely, even a significant reduction of Tet-resistant ETEC in the stationary phase within 12 required a 4 × MIC concentration of CNMs; after 24 h, bacteria tended to regrow. Although the bactericidal activity decreased in the different phases of Tet-resistant ETEC, different concentrations of CNMs still had great antimicrobial activity.

CNMs kill anti-Tet-ETEC under conditions that mimic the gastrointestinal tract. The antibacterial activity of CNMs was evaluated by simulating the real situation of the animal gastrointestinal tract (**Figures 1K–M**). Therefore, we prepared synthetic gastroenteric fluid that mimics the real gastrointestinal environment. The pH of the synthetic gastric juice was adjusted to 1.5 or 2 to simulate the normal range of the stomach. In the natural environment, the bacteria obtained by animals were in the stationary phase, so 5 × 10^8^ cfu/ml stationary-phase bacteria were inoculated into synthetic gastric juice. At pH 1.5, due to the acidic environment, all bacteria were removed after 1 h (data not shown). Therefore, the pH was increased to 2 (**Figure 1K**), and the results showed that 0.05% and 0.1% CNMs could inhibit the bacteria completely after 1 h, respectively. Compared with the control group, 0.025% of CNMs significantly killed Tet-resistant ETEC, and an approximately 6-log reduction was observed within 1 h of CNM treatment. To a great extent, CNMs could kill antibiotic-resistant microorganisms from the stomach; thus, oral administration of CNMs could kill a large number of Tet-resistant ETEC, and smaller amounts of Tet-resistant ETEC could pass into the small intestine.

Subsequently, we examined the bactericidal activity of CNMs in intestinal fluid with 5 × 10^8^ cfu/ml Tet-resistant ETEC at the stationary phase (**Figure 1L**). In the simulated small intestinal environment, 0.1% and 0.2% CNMs reduced the bacteria to non-detectable numbers within 1 h. Compared to the control group, 0.05% CNM significantly killed Tet-resistant ETEC, and an approximately 7-log reduction was observed within 1 h of CNM treatment. These data suggest that oral administration of CNMs could eliminate pathogens and have great potential to increase human and animal health through the prevention and treatment of antibiotic-resistant bacterial infections.

Antibiotic-resistant bacteria are able to cause gastrointestinal diseases and even death in humans and animals by contaminated food and water (25, 34). Thus, we tested the efficacy of CNMs against the inoculated stationary phase of Tet-resistant ETEC in different food samples (**Figure 1M**). We found that when the three products, skim milk (SKM), mincemeat supernatant (SMS), and sterilized egg yolk (SEY), contained 8 × MIC CNMs and Tet-resistant ETEC concentrations were 10^3^ cfu/ml, no viable bacteria were detected after 2 h (0 cfu in three 100-µl product samples, data not shown). When 10^5^ cfu/ml pathogenic ETEC was tested for 2 h, we found that Tet-resistant ETEC cultures inoculated into the three biological products (SKM, SEY, and SMS) with 8 × MIC CNMs were significantly decreased compared with the SSW group. An approximately 10^3^ cfu/ml reduction in Tet-resistant ETEC was obtained for SKM, SEY, and SMS.

### Mode of Action of CNMs Against Tet-Resistant ETEC Study

#### CNMs Disrupt the Membrane Integrity of Tet-Resistant ETEC

**Figure 2:**
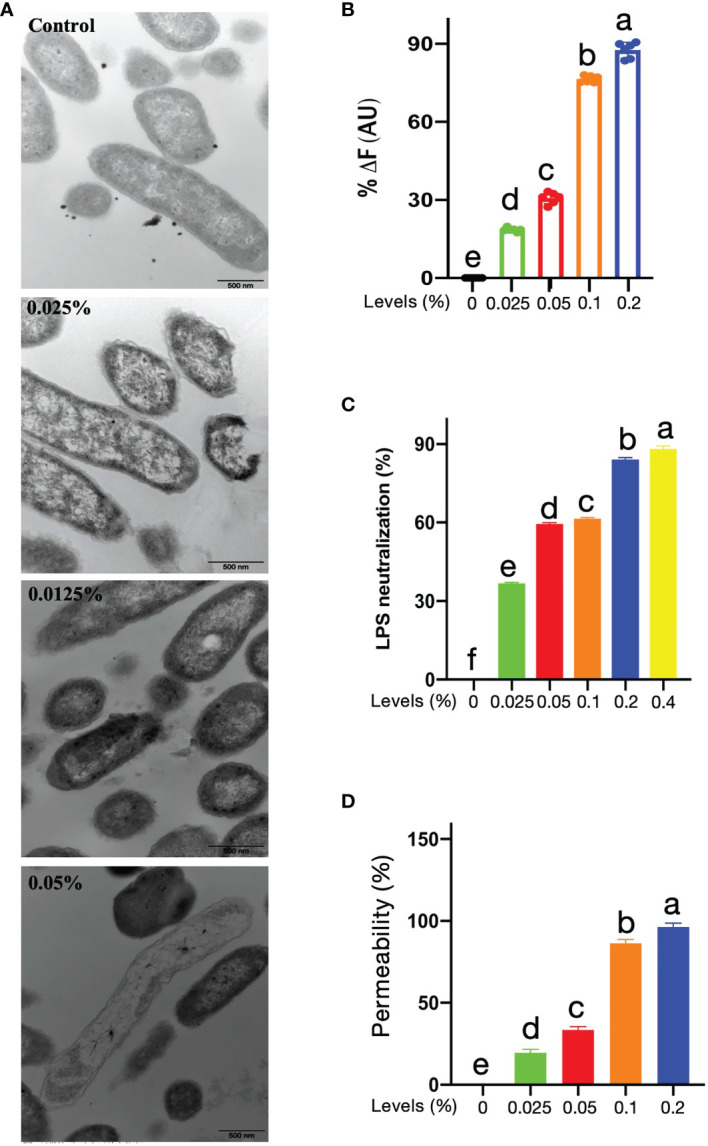
Potential of mechanism of CNMs. **(A)** Transmission electron microscopy micrographs of Tet-resistant ETEC treated with CNMs. Stationary-phase bacteria cells at around 10^6^ cfu/ml were treated with different concentrations of CNMs for 1 h. The change of cytoplasmic membrane was determined. Control, untreated; 0.025% of CNMs, 1 × MIC; 0.05% of CNMs, 2 × MIC; 0.1% of CNMs, 4 × MIC. Images are presented in three independent experiments. **(B)** LPS-binding affinity and **(C)** endotoxin neutralization of the CNMs at variant levels were tested by the BODIPY-TR cadaverine fluorescent dye displacement assay and chromogenic limulus lysate, respectively. Data are presented as the means ± standard error of three independents trials. **(D)** Outer membrane permeabilization of Tet-resistant ETEC treated with different levels of CNMs was determined using N-phenyl-1-naphthylamine (NPN) dye. The fluorescence was measured to indicate the permeability. Data are presented as the means ± standard error of three independents trials. **(B–D)** values that different lowercase are significantly different (P < 0.05) in each bar graph.

The TEM has been applied to directly determine the effects of antimicrobials on the integrity of bacteria and to witness ultrastructural changes. The results showed that after treatment with 1, 2, or 4 × MIC CNMs, the plasma membrane integrity of Tet-resistant ETEC cells was adversely affected. As shown in **Figure 2A**, the cytoplasm of untreated anti-Tet-ETEC cells was dense, the cytoplasm was surrounded by a complete cell membrane, and some clear cytoplasmic bands could be seen. CNMs treatment groups at 1 and 2 × MIC significantly destroyed the Tet anti-ETEC cell membrane, released intracellular contents, and exhibited zona pellucida in the cytoplasm. After 4 × MIC CNMs treatment, Tet-resistant ETEC cells were encapsulated in the damaged membrane. CNMs membrane deformation at 2 and 4 × MIC was small, the cytoplasmic membrane was invisible, the electron density was uneven, and the electron light region and cell wall became thinner. The total leakage of cytoplasmic contents was also evident.

#### Capacity of LPS Binding of CNMs

A previous study demonstrated that CNs interact with LPS, which results in bactericidal activity (35). Thus, we evaluated the LPS-binding capacity of CNMs (**Figure 2B**). A displacement assay with fluorescence was conducted to assess the LPS-binding affinity of CNMs. The results showed that CNMs were able to bind to LPS in a dose-dependent manner; 0.1% CNMs resulted in an increased fluorescence intensity of more than 70%, and the increase in the fluorescence intensity induced by 0.2% CNMs was approximately 88%.

We further investigated the effects of CNMs on endotoxin neutralization (**Figure 2C**). We found that CNMs could also neutralize endotoxin in a concentration-dependent manner, and the efficiency of neutralizing endotoxin of CNMs increased rapidly with increasing CNMs concentration. CNMs (0.05%) resulted in approximately 60% neutralization when the CNM concentrations were 0.2% and 0.4%, respectively, with endotoxin incubation, and the efficiency of neutralizing endotoxin was more than 84%.

#### Outer Membrane Permeabilization

A previous study demonstrated that CNs interact with outer membrane protein A (OmpA), which results in bactericidal activity (35). Moreover, peptides can damage the bacterial cell membrane and result in bacterial death (36–38). The damage ability of CNMs on the outer membrane of Tet-resistant ETEC bacteria was studied by using NPN dye (**Figure 2D**). By measuring the ratio of CNMs-induced fluorescence to the fluorescence generated by the membrane active compound polymyxin B, the CNMs-induced fluorescence increase was converted into outer membrane permeability, which was considered 100% membrane permeability. As shown in **Figure 2C**, the results were similar to the binding power of LPS. CNMs is concentration-dependent on membrane permeability. CNMs has strong membrane penetration, resulting in the death of Tet-resistant ETEC. The membrane permeability of CNMs increases rapidly with increasing CNMs concentration. CNMs (0.05%) resulted in approximately 34% membrane permeabilization when the CNMs concentration was 0.1% and 0.2% with Tet-resistant ETEC incubation, which led to 86.29% and 95.13% membrane permeabilization, respectively.

#### CNMs Exert Antioxidant Capacity

**Figure 3:**
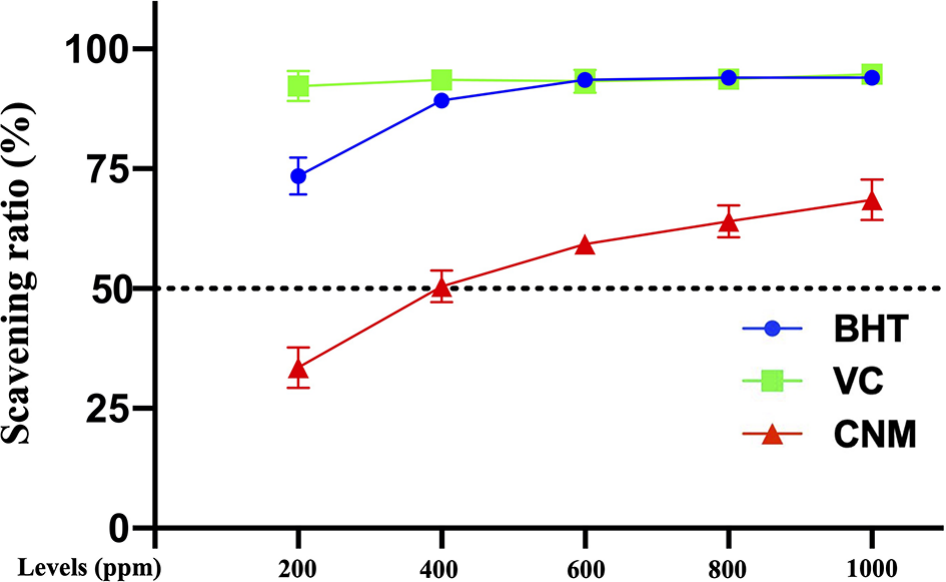
The free radical scavenging ratio was determined by the DPPH method. CNMs at different MIC levels were mixed with DPPH solution. The mixtures were shaken well and put in the dark at room temperature for 30 min. Then absorbance was determined using a Q-6 UV-Visable Spectrophotometer at 517 nm. BHT and VC were used as positive control. Data are presented from two independent experiments.

Antimicrobial- or nanoparticle-induced oxidative stress, such as excessive free radicals, has become a major concern in the application of food, agriculture, and medical industries. DPPH, officially known as 2,2-diphenyl-1-picric acid hydrazine, is a cell-permeable substance. DPPH reacts with antioxidants or reducing compounds to produce the corresponding hydrazine DPPH. The determination of the DPPH scavenging ratio is a rapid and stable method to evaluate the antioxidant activity of antimicrobials. Thus, the antioxidant capacity of CNMs was detected by the DPPH method, and BHT and VC were used as positive controls. As shown in **Figure 3**, the results showed that the antioxidant ability of CNMs was concentration-dependent, and approximately 0.04% CNMs resulted in a 50% DPPH free radical scavenging ratio (**Table S3**). Although the DPPH free radical scavenging ratio of CNMs was lower than that of BHT and VC, CNMs still exerted excellent antioxidant ability by DPPH decreasing free radicals, indicating that CNMs may effectively reduce oxidative stress.

### Anti-Inflammatory of CNMs

#### CNMs at the Antibacterial Level Did Not Induce Cytotoxicity to RAW264.7 Cells

**Figure 4:**
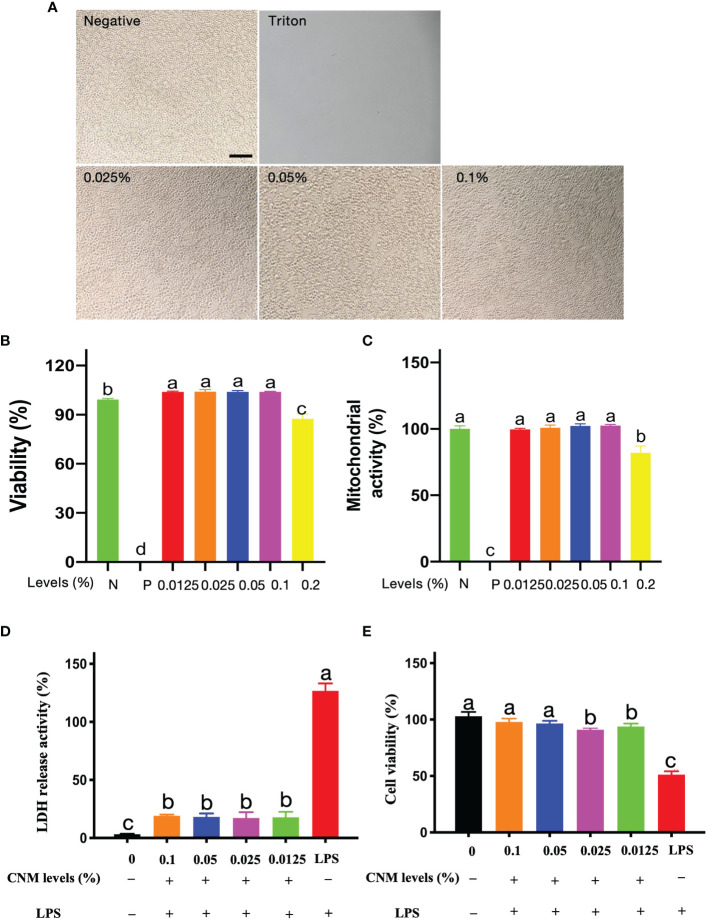
Cytoxicity risk assessment of CNMs at various concentrations. **(A)** Cell morphology was observed after treatment with CNMs. Mouse RAW264.7 cell morphology after treatment with 0%, 0.025%, 0.05%, and 0.1% CNMs for 24 h. Triton X-100 was used as a positive control. Morphology change of cells was measured. The scale bar indicates 200 µm. Data are presented from two independent experiments. **(B, C)** The cytotoxic activity of CNMs toward RAW264.7 cells. Cells were treated with various concentrations of CNMs for 24 h, then cell viability was determined by LDH assay **(B)**; mitochondrial activity was measured by MTT assay **(C)**. Positive control was Triton X-100. Data shown as the mean ± standard error of three independent experiments. Means with different lowercase letters indicate significance (*p* < 0.05). N: negative control; P: positive control. **(D, E)** CNMs inhibited LPS-induced cytotoxicity to RAW264.7 cells. CNMs at different concentrations were added to the medium and cultured for 24 h, then 1 µg/ml LPS was applied to treat cells and cultured for 8 h. LDH and MTT assays were applied to evaluate the anti-LPS-induced cytotoxicity of CNMs. LDH release assay **(D)** and MTT assay **(E)** of RAW264.7 cells treated with CNMs at various concentrations. Data are indicated as the mean ± standard error from six biological replicates. Means with different lowercase letters indicate significance (*p* < 0.05).

Based on LPS binding and neutralization analysis, we further tested the anti-inflammatory responses of CNMs against LPS-induced macrophage inflammation. To be able to apply in the host, it is essential to ensure that CNMs do not cause toxicity in the intestine at working concentrations. Risk assessment is the first step to examine whether CNMs damage cell lines by visualizing cell morphology and viability after antimicrobial treatment. We investigated the after treatments with 0.025%, 0.05%, and 0.1% CNMs representing 1 ×, 2 ×, and 4 × MIC. As shown in **Figure 4A**, different concentrations of CNMs did not alter the morphology of RAW264.7 cells compared with the control treatment, and irregular and dead cells were not detected. CNM treatments did not damage any RAW264.7 cells or cause cell death, indicating that CNMs have no toxicity to cells at working levels.

Cell morphology and viability visualization are not enough evidence to demonstrate that CNMs are non-toxic to the cells. Consequently, cell viability and mitochondrial activity were further measured by LDH and MTT assays, respectively. CNMs were employed at 0.5 ×, 1 ×, 2 ×, 4 ×, and 8 × MIC levels. The results are presented as the viability percentage of cells compared with the negative control (100%). Triton X-100 was applied as a positive control. As shown in **Figure 4B**, 0.5 ×, 1 ×, 2 ×, and 4 × CNMs did not affect cell viability. Compared with the NC group, 0.5 ×, 1 ×, 2 ×, and 4 × concentrations of CNMs significantly increased microphage viability. However, 8 × MIC moderately decreased the viability of RAW264.7 cells by approximately 10% compared to that of the NC cell group.

We tested whether CNMs affect the mitochondrial activity of RAW264.7 cells by MTT assay **(** **Figure 4C** **)**. The cells challenged with various concentrations of CNMs did not decrease the metabolic activity of RAW264.7 cells compared with the control group, even at 0.1%, equivalent to 4 × MIC. However, when CNMs were incubated with the cells at 8 × MIC, the mitochondrial activity significantly decreased by approximately 20%. These results show that CNMs, even at MICs higher than the MIC level, do not cause cytotoxicity against RAW264.7 cells.

#### CNMs Ameliorate LPS-Induced Cytotoxicity to RAW264.7 Cells

Because CNMs non-toxicity alone results in mouse RAW 264.7 macrophages, an LDH assay was conducted to determine the effects of CNMs with LPS in mouse RAW 264.7 macrophages (**Figures 4D, E** **)**. The data show that LPS treatment in the absence of CNMs induced a higher release of LDH at 8 h than that in the control groups, indicating that LPS damaged the RAW264.7 cell membrane. However, CNM (0.5 ×, 1 ×, 2 ×, and 4 × MIC) treatment significantly decreased LPS-induced LDH release, which was similar to the control group (**Figure 4D**). We further tested the effects of CNMs on cell viability after LPS challenge (**Figure 4E**). We found that LPS treatment significantly decreased cell viability compared to that of the control groups, indicating that LPS caused cell death. However, CNM (0.5 ×, 1 ×, 2 ×, 4 × MIC) treatment significantly improved the LPS-induced decrease in cell viability, which was similar to the control group.

These findings indicate that LPS disrupted the mouse RAW264.7 macrophage cell membrane and led to cell death, but different working concentrations of CNMs significantly ameliorated LPS-induced cytotoxicity to RAW264.7 cells. Mechanistically, CNMs were neutralized and bound to LPS. Notably, the engineered CNMs based on the peptide and chitosan nanoparticles also decreased LPS-induced cytotoxicity.

#### CNMs Inhibit the Inflammatory Response by Downregulating LPS-Stimulated Proinflammatory Cytokines

**Figure 5:**
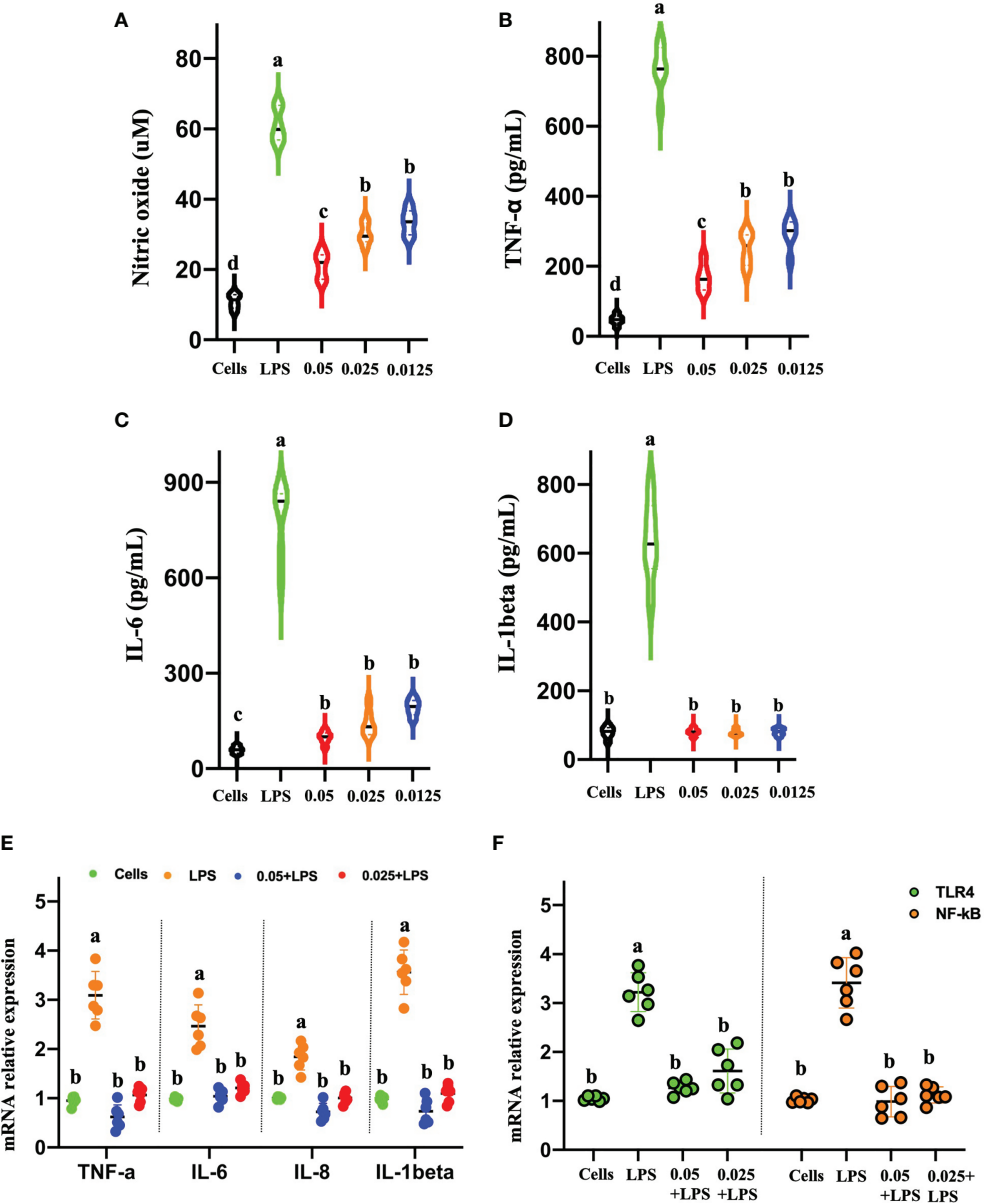
CNMs attenuated proinflammatory responses in LPS-challenged RAW264.7 cells. RAW264.7 cells were treated with 1 µg/ml LPS and incubated with CNMs at different concentrations (0.0125%, 0.025%, and 0.05%). Supernatant was collected at 24 h after culture, and the contents of nitric oxide **(A)** and tumor necrosis factor-α **(B)**, interleukin (IL)-6 **(C)**, and IL-1β **(D)** were detected by ELISA. **(E, F)** The proinflammatory cytokines mRNA expression levels of TNF-α, IL-6, IL-1β **(E)**, TLR4, and NF-κB **(F)** were determined by qPCR. Data are indicated as the mean ± standard error from six biological replicates. Means with different lowercase letters indicate significance (*p* < 0.05).

We are encouraged by the ability of CNMs to neutralize and bind LPS by inhibiting LPS-induced inflammatory responses. To address this issue, we discussed the effects of CNMs on the production and expression of the proinflammatory cytokines secreted by NO, TNF-α, IL-6, and IL-1β by LPS-induced mouse RAW264.7 macrophages (**Figure 5**). Our results showed that LPS stimulation significantly increased NO levels compared to the control group, but treatment with CNMs (0.5 ×, 1 ×, and 2 × MIC) markedly reduced the NO level induced by LPS, as shown in **Figure 5A**. Furthermore, we measured TNF-α, IL-6, IL-8, and IL-1β production and expression by ELISA and qPCR methods (**Figures 5B–E**). CNMs significantly decreased the secretion of TNF-α, IL-6, and IL-1β and reduced the expression of TLR4, NF-κB (**Figure 5F**), TNF-α, IL-6 IL-8, and IL-1β in mouse RAW264.7 macrophages compared with those in cells infected with LPS only.

**Figure 6:**
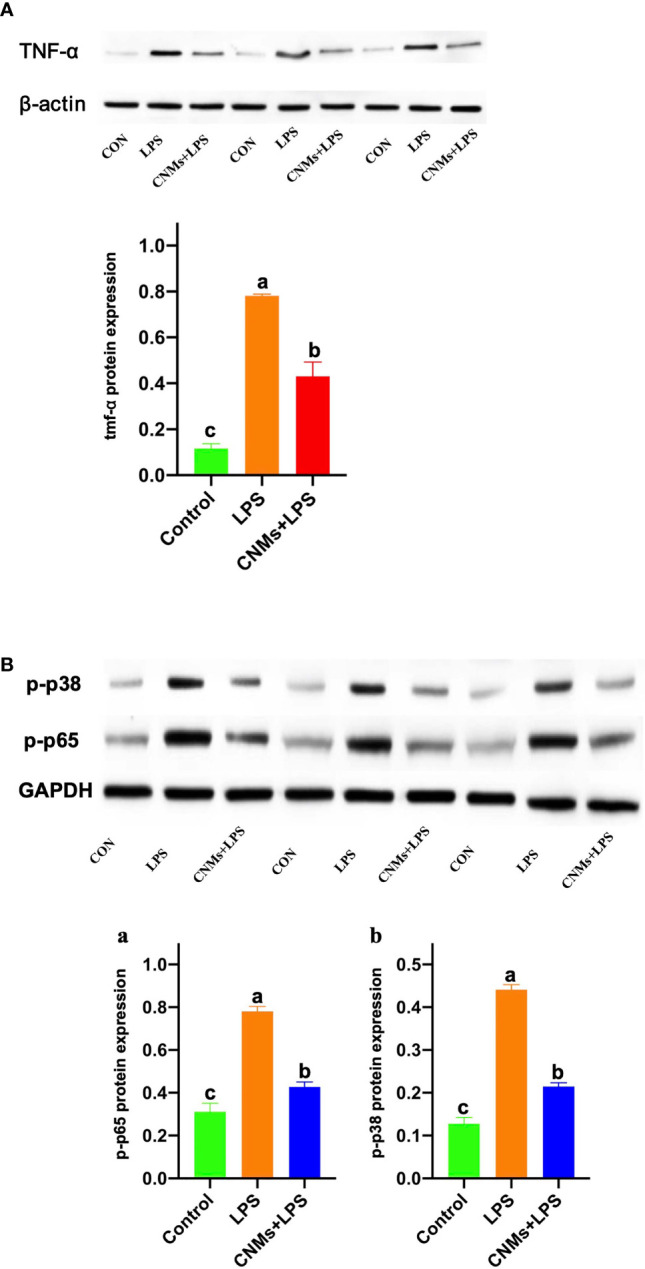
Protein expression of proinflammatory cytokines was determined by Western blotting. Cells were treated with 1 µg/ml LPS and incubated with CNMs at 0.05% concentrations. Cells in different treatments were collected at 24 h after culture. The TNF-α, p-p38 MAPK, and p-NF-κB protein expression was analyzed by Western blotting in RAW264.7 cells. RAW264.7 cells were pretreated or not with CNMs for 24 h and then challenged with LPS. Cells were collected after LPS challenge. **(A)** The abundance of TNF-α. **(B)** The abundance of phosphorylated NF-κB **(a)** and p-p38 **(b)**. The results were indicated as means ± standard error. Different superscript lowercase letters among groups indicate significant differences (*p* < 0.05).

**Figure 7:**
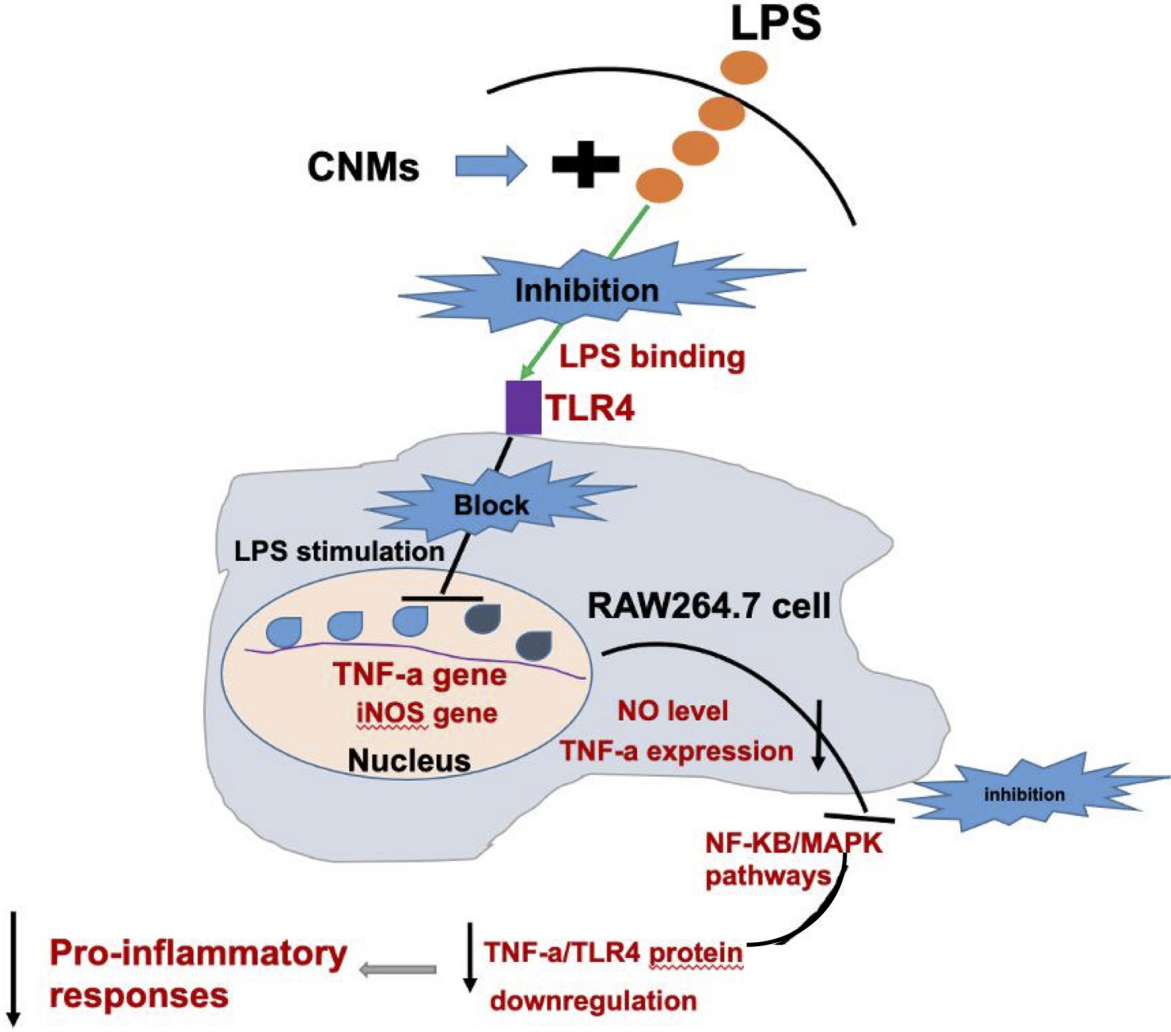
Schematic diagram of a potential mechanism by which CNMs suppress LPS-induced inflammatory responses.

Western blot results demonstrated that LPS treatment resulted in increased TNF-α, NF-κB, and mitogen-activated protein kinase (MAPK) protein expression (**Figure 6**). CNMs significantly decreased the TNF-α (**Figure 6A**), NF-κB (**Figure 6Ba**), and MAPK (**Figure 6Bb**) abundances induced by LPS challenge. Critically, 0.05% CNM treatment reduced the proinflammatory cytokines to almost the same level as that in the control group. The results of the present study preliminarily showed that engineered CNMs based on peptides and chitosan nanoparticles have significant anti-inflammatory activity by affecting the NF-κB and MAPK pathways to decrease proinflammatory cytokines (**Figure 7**).

## Discussion

In the current study, we demonstrated that CNMs engineered with chitosan nanoparticles and MccJ25 exert excellent bactericidal activity against foodborne and intestinal pathogens, including Tet-resistant ETEC, by disrupting the cell membrane and binding LPS. Furthermore, CNMs did not cause RAW264.7 cell cytotoxicity or improve LPS challenge-induced RAW264.7 cell toxicity risk and had no side effects. Anti-inflammatory responses indicate that CNMs could ameliorate LPS-induced inflammation by decreasing proinflammatory cytokine levels *via* the TLR4-NF-κB and p38 MAPK pathways. CNMs, a novel nanoantimicrobial polymer, could be used as antimicrobial/antiendotoxin agents to treat inflammatory bowel disease caused by LPS or pathogens.

In the post-antibiotic era, a large number of clinical ARMs, including foodborne pathogens and intestinal pathogenic bacteria, have been on a rapid outbreak in recent years (12, 39). In addition, the subsequent spread of drug-resistant strains from non-human sources in the environment, such as livestock or edible animals, using antibiotics similar to those used to treat human infections is a major factor in the expansion of antibiotic resistance (40). Food spoilage is caused by pathogenic bacteria (36). In this regard, there is no doubt that infections caused by bacterial infections are the biggest killers of health (7). Therefore, new antimicrobial strategies and novel types of agents are required to tackle the life-threatening problems of bacterial infection and drug resistance.

Nanomaterials as antimicrobials are raising a significantly attractive strategy for preventing bacterial infections and have substantially been applied in food safety, medicine, and agriculture as conventional antimicrobial stewardship. Nanomaterials possess potent drug loading ability and can provide an efficient platform for the successful application of engineered materials to effectively combine antibacterial agents against ARM bacterial infections. Currently, nanomaterials, especially antimicrobial polymeric nanomaterials (APNs), play crucial roles in antimicrobial domains and exhibit enormous potential as enhancers or therapeutic or preventive agents to treat serious ARM bacterial infections (17–21, 27).

In a previous study, we designed and engineered CNMs formed by AMP MccJ25 and CNs. We demonstrated that engineered CNMs could be excellent novel antimicrobial agents against pathogens because of their excellent antibacterial activity, low toxicity, and lack of drug resistance (32). Based on our previous study, we further conducted a comprehensive antimicrobial assay to evaluate the effectiveness of the bactericidal activity of CNMs. First, we tested the MIC value of CNMs against different pathogens (**Table S2**), and we found that CNMs had the lowest MIC (0.025%) toward Tet-resistant ETEC among all the tested bacteria. For the MICs of other pathogens, the results were consistent with our previous study that CNMs had 0.05% MIC against MRSA, *E. coli* O157, and swine sources *E. coli* K88 (32). Time-killing curves based on the MICs were conducted to further confirm that CNMs have strong antimicrobial activity against clinically foodborne pathogens and intestinal pathogens. Meanwhile, Tet-resistant ETEC as the indicated bacterium was tested by live/dead assay staining with propidium iodide. Consistent with our *E. coli* O157 previous study (32), CNMs disrupt the cell membrane to induce Tet-resistant ETEC death. In addition, it has been reported that CNs and AMP can break the bacterial cell membrane to kill pathogens (27, 29, 30, 36, 37); thus, CNMs perfectly combine the mode of action of CNs and AMP to exert greater antibacterial ability.

CNMs killed pathogens, similar to other ANPs. Liu et al. (41) reported that a series of nanoparticles self-assembled by amphiphilic peptide (CG3R6TAT) have good bactericidal ability and significantly reduce the colonization of *Staphylococcus aureus* in the intestinal tract of mice. Lam et al. (42) developed polypeptide polymer nanoparticles composed of lysine and valine residues. They not only have good antibacterial activity against gram-negative bacteria but also effectively kill drug-resistant *E. coli* and multidrug-resistant pathogens, and the antibacterial mechanism is multifaceted, including destroying the integrity of the bacterial outer membrane and plasma membrane.

Both MccJ25 and CNs have shown that bactericidal activities are not related to bacterial cell wall synthesis but disrupt bacterial cell walls (25, 30). Although engineered CNMs and ANPs are formed by AMP MccJ25 and CNs, we do not know whether the bactericidal activities of CNMs are similar to those of AMP MccJ25 and CNs. We conducted bacterial cells at different growth phases to test this hypothesis. In addition, when foodborne bacterial pathogens enter the host cell, the number of bacteria is usually low, but the pathogen will replicate to a higher number in its niche, indicating the existence of infectious diseases. To evaluate whether CNMs can also be used to treat diseases in the late stage of the infection cycle, we simulated the late stage of infection and used a large number of 5 × 10^8^ cfu/ml bacteria. Our findings indicated that CNMs can completely kill different growth phases of Tet-resistant ETEC. However, compared with log-phase pathogens, stationary-phase cells were more resistant to CNMs, but significant reductions were still achieved. Moreover, CNMs have stronger bactericidal activity in early-log-phase Tet-resistant ETEC than in the late-log phase. These results are also similar to AMP MccJ25 and CNs (25, 30). In natural environments, most intestinal diseases are caused by stationary-phase bacteria. Stationary-phase bacteria were more resistant to antimicrobial treatments, including high pressure, high temperature, and fungicides, than digital phase cells. Related studies also showed that compared with logarithmic bacteria, phasing bacteria had a complete outer membrane, a high protein-to-fat ratio, a low unsaturated fatty acid content, and a thicker cell wall (43). Therefore, phasing bacteria were stable. In conclusion, although bacteria in food, gut, or water may be in the quiescent phase due to nutritional restriction and stress, it is feasible to reduce pathogenic cells in the quiescent phase with higher concentrations of antimicrobial agents or with longer culture times.

Microcins and chitosan micro/nanoparticles as natural and promising antimicrobial agents have attracted interest in the application of inhibiting numerous food spoilage and pathogenic bacterial infections, including food, medicine, and veterinary domains (13, 29, 44). In our previous study, we showed that CNs, MccJ25, and CNMs are stable at low-pH and high-temperature conditions (32). The application of CNs and MccJ25 as food preservatives extends the shelf life of foods, reduces the risk of transmission of foodborne pathogens *via* the food chain, improves food spoilage, and allows the use of less serious treatments during food processing without compromising food safety. In this study, CNMs did not halt the growth of Tet-resistant ETEC but did significantly reduce their number on different biological food products, including egg yolk, milk, and meat.

However, these data presented in this study demonstrate the potential applications of CNMs to target pathogens, and any application of antimicrobial agents in food, medicines, or livestock. When taking antibiotics orally, we encounter some obstacles, which may affect their stability and biological activity. Oral or *in situ* proteins are rapidly inactivated or degraded by proteolytic enzymes such as pepsin, trypsin, and chymotrypsin in the stomach and small intestine (25, 30, 37). It is important to clarify any factors that may interfere with its activity, such as intestinal pH or protease. In this paper, we examined the degradome of CNMs using SGF and SIF *in vitro* models of digestion associated with antibacterial assays. Subsequently, simulated gastrointestinal conditions *in vitro* were employed to evaluate the antimicrobial activity of CNMs, to further understand the fate and stability of oral CNMs. Because CNMs need to kill pathogens in the intestine, CNMs should maintain their bactericidal properties without being destroyed or degraded by gastrointestinal bile salts or digestive enzymes. In SGF and SIF, 0.05% CNM was sufficient to eliminate 5 × 10^8^ cfu/ml cells in 60 min. We have demonstrated that engineered CNM exhibits pH and protease resistance while maintaining high antimicrobial activity in the intestine and simulated environment. This observation is consistent with previous reports that CM has an enhanced bactericidal effect under acidic conditions, while MccJ25 is very stable under extreme conditions due to its lasso topology (25, 30, 45, 46).

CNMs could break bacterial cell walls and damage the cell membrane to kill pathogenic bacteria. In a previous report, CNs and MccJ25 were reported to disrupt bacterial cell walls and damage the cytoplasmic membrane, causing cell death (25, 30). Additionally, it has been indicated that CNs interact with LPS, which causes bactericidal antimicrobial activity (35). We assumed that CNMs could take antimicrobial advantages of CNs and MccJ25. To test this regard, we further investigated the antimicrobial mechanisms of CNMs toward Tet-resistant ETEC. CNM has the property of rapid bacterial killing, which is considered characteristic of ANP, AMP, or membrane-destroying antibiotics. However, the mode of action of CNM on pathogens, especially drug-resistant microorganisms, is not fully understood. Endotoxins act as the first barrier of penetration for gram-negative bacteria, especially *E. coli*, and block the action of most antibiotics (47, 48). The lipopolysaccharide-binding and neutralization activities of CNMs were studied for the first time. These results indicate that CNMs can effectively exert the binding affinity and neutralizing activity of LPS, thus blocking the biological effects of endotoxin. It is known that the membrane interaction between antibacterial agents and polyanionic lipopolysaccharides is the initial process after which antimicrobials penetrate the cell membrane (36, 37). CNMs penetrates the bacterial outer membrane in a dose-dependent manner. In addition, CNMs that destroy the outer membrane can be trapped in the periplasmic space, destroy the plasma membrane of cells, lose membrane potential, and cause bacterial death. Therefore, transmission electron microscopy further confirmed the influence of CNMs on the plasma membrane. Lysis of Tet-resistant ETEC caused by CNMs was observed. The cytoplasmic membrane, as well as the release of fluid and debris, thinned, broke, and disintegrated. In the study of membrane permeability, CNMs showed a stronger ability to interfere with bacterial membrane permeability, especially to destroy the cytoplasmic membrane, which made CNMs have higher bactericidal activity.

Based on the binding affinity and neutralizing activity of LPS, our hypothesis is supported that this combination has strong anti-inflammatory activity. CNMs have strong antibacterial activity, are non-toxic, and are safe. Although we have shown that CNMs do not cause cytotoxicity in HEK293T and Caco-2 cells (32), the cytotoxic activities of different cell types are not clear. Thus, in the present study, CNMs were first subjected to cytotoxicity assays in mouse macrophage RAW264.7 cells. We found that CNMs did not damage the cell membrane and did not disrupt mitochondrial activity, indicating that CNMs do not induce cytotoxicity in RAW264.7 cells. Notably, CNMs significantly decreased LPS-induced RAW264.7 mouse macrophage damage and decreased mitochondrial activity. Before application of antimicrobial agents, different types of assays should be used for *in vitro* cytotoxicity evaluation because the reasons for different cytotoxic activities in different cell types are yet to be known (13). Risk assessments need to be evaluated because higher concentrations of antimicrobials can permeabilize the membrane, as well as the type of eukaryotic cells and their metabolic activity and the type of assay. Our study preliminarily showed that CNMs suppress intestinal inflammation.

In the present study, the production and expression of the proinflammatory cytokines NO, TNF-α, IL-6, IL-1β, and IL-8 in mouse RAW264.7 macrophages were evaluated. LPS can be released when pathogenic bacteria, especially *E. coli*, are killed in the host and induce intestinal inflammatory responses. After ETEC invades the intestine of the host, it begins to rapidly colonize, rejuvenate and reproduce, and secrete enterotoxin and LPS, resulting in intestinal inflammation (47–49). Intestinal inflammation is a chronic inflammatory disease that destroys the immune balance through an increase in intestinal proinflammatory cytokine secretion (50, 51). The innate immune molecule TLR4 on intestinal cells recognizes LPS and activates downstream NF-κB, and other signaling pathways which promote downstream proinflammatory factors (TNF-α, IL-6, etc.), and is secreted and expressed in large quantities, which induces an excessive intestinal inflammatory response in the host and causes inflammatory injury (52–55). Similar to inflammation, cells become activated and secrete large amounts of NO, enhancing damage and tissue damage. Reducing NO production may be a new strategy to fight inflammatory diseases. Stimulated macrophages also produce a large number of proinflammatory cytokines that participate in the upregulation of inflammation, which may lead to diseases such as IBD (56–60). Therefore, reducing the proinflammatory response is very important to alleviate inflammatory disease. In this study, we found that CNMs more effectively inhibited the production of proinflammatory cytokines, mRNAs, and proteins, such as TNF-α, IL-6, IL-1β, IL-8, TLR4, p-NF-κB, and p-p38. TLR4 and NF-κB are critical regulatory molecules. Thus, NF-κB and MAPK are regarded as crucial active factors in regulating inflammatory gene expression in the present study (as shown in **Figure 7**).

Additionally, in recent years, chitosan and its derivatives, including chitosan micro/nanoparticles, have attracted increasing attention because of their excellent antioxidant activity (61, 62). Oxidative stress is able to induce an inflammatory response, and IBD, for instance, LPS-induced oxidative stress. The occurrence of oxidative stress indicates that the ability of body tissue to scavenge free radicals is reduced. Inhibition of NF-кB activity can alleviate the damage response caused by oxidative stress (63, 64). Moreover, antimicrobial or nanoparticle toxicity related to the induction of oxidative stress is becoming a major concern in food, medicine, and agriculture applications. In this study, we found that CNMs exert the scavenging capacity of DPPH free radicals, indicating that CNMs could possess antioxidative activity. Additionally, the effects of CNMs on reactive oxygen species generation need to be further studied.

These observations suggest that the nanoengineered APNs and CNMs of AMP and CNs may be attractive antimicrobial candidates for the treatment of bacterial infections for food safety, medicine, and agriculture.

## Conclusion

In this study, CNMs, engineered by AMP and CNs, showed excellent bactericidal activity against different clinically foodborne pathogens and intestinal pathogenic bacteria, including tetracycline-resistant ETEC, by comprehensive antimicrobial activity determination. A potential antimicrobial mechanism indicated that CNMs efficiently exert LPS binding activity, membrane disruption, and membrane permeability ability to Tet-resistant ETEC. The membrane-breaking mechanism of the CNMs was further confirmed by transmission electron microscopy. In addition, CNMs show anti-endotoxin, immunomodulatory, and anti-inflammatory activities by binding to *E. coli* LPS. Lipopolysaccharides induced the increase in proinflammatory cytokines in RAW264.7 macrophages which were inhibited, and the cytotoxic activity was reduced. These findings provide a promising strategy for CNMs as a potential therapeutic/preventive agent for bacterial infection and suggest that CNMs can control pathogenic pollution in the food industry. However, the additional properties of CNMs as food additives or antimicrobial agents in the treatment of animal model infections need to be further studied. In addition, although antibiotics are widely used in food, medical treatment, animal husbandry, and agriculture, their safety still needs to be taken seriously, such as *in vitro* and *in vivo* toxicity data.

## Data Availability Statement

The datasets generated and presented in the study are included in the article/**Supplementary Material**. Further inquiries can be directed to the corresponding author.

## Author Contributions

YH, CY, and HS conceived and designed the study. YH and CY performed the antimicrobial and cell experiments. SM performed the antioxidant activity experiment. YH and CY analyzed the data. YH wrote the manuscript. CY and HS contributed to the revision of the manuscript. All authors contributed to the article and approved the submitted version.

## Funding

This study was supported by Talent Introduction Projects of Hebei Agricultural University (YJ2020005) and Natural Science Foundation of Hebei Province (C2021204161).

## Conflict of Interest

The authors declare that the research was conducted in the absence of any commercial or financial relationships that could be construed as a potential conflict of interest.

## Publisher’s Note

All claims expressed in this article are solely those of the authors and do not necessarily represent those of their affiliated organizations, or those of the publisher, the editors and the reviewers. Any product that may be evaluated in this article, or claim that may be made by its manufacturer, is not guaranteed or endorsed by the publisher.
