# Supplementary material for: A Novel Polymeric Nanohybrid Antimicrobial Engineered by Antimicrobial Peptide MccJ25 and Chitosan Nanoparticles Exerts Strong Antibacterial and Anti-Inflammatory Activities
Source: Front Immunol. 2022 Jan 19;12:811381. doi: 10.3389/fimmu.2021.811381 (PMC8807516; doi:10.3389/fimmu.2021.811381)
Supplement: Supplementary file 1 [file Table_1.docx]

**Supplementary Materials**

**Running Title: Polymeric Antimicrobial for Treating Intestinal Inflammation**

**A Novel Polymeric Nanohybrid Antimicrobial Engineered by Antimicrobial Peptide MccJ25 and Chitosan Nanoparticles Exert Strong Antibacterial and Anti-Inflammatory Activities**

*Yu Haitao^1,2^, Chen Yifan^2^, Sun Mingchao^3^ and Han Shuaijuan^2^*

*^1^Institute of Systems Biomedicine, Department of Immunology, School of Basic Medical Sciences, Beijing Key Laboratory of Tumor Systems Biology, Peking University Health Science Center, Beijing, 100191, China;*

^2^*College of Animal Science and Technology, Hebei Agricultural University, Baoding, 071000, Hebei province, P.R. China;*

^3^*State Key Laboratory of Animal Nutrition, Institute of Animal Sciences of Chinese Academy of Agricultural Sciences, Beijing 100193, P.R. China;*

**Corresponding authors: Han ShuaiJuan, Associate Professor*

*E-mail: hansjuan@163.com*

**Materials and methods**

**Table S1 qRT-PCR primer sequences applied in this study.**

| Genes |  | Sequence (5'-3') | Size (bp) | NCBI Gene ID |
| --- | --- | --- | --- | --- |
| *GAPDH* | Forward | GAGAAACCTGCCAAGTATGATGAC | 212 | NM_017008.3 |
|  | Reverse | TAGCCGTATTCATTGTCATACCAG |  |  |
| *TNF-α* | Forward | CCACGCTCTTCTGTCTACTG | 169 | NM_010851.2 |
|  | Reverse | ACTTGGTGGTTTGCTACGAC |  |  |
| *IL-6* | Forward | GAGTCACAGAAGGAGTGGCTAAGGA | 106 | NM_031168.1 |
|  | Reverse | CGCACTAGGTTTGCCGAGTAGATCT |  |  |
| *IL-1β* | Forward | GGACAGCCTGTTACTACCTGACACATT | 239 | NM_031512 |
|  | Reverse | CCTAGGAAACAGCAATGGTCGGGAC |  |  |
| *IL-8* | Forward | TCTCTGGCCTTGGAACATAGTCT | 176 | NM_003361958 |
|  | Reverse | TTCGATGCCAGTGCATAAATA |  |  |
| *TLR4* | Forward | GTTTGCTCAGGATTCGAGGC | 160 | AF185285.1 |
|  | Reverse | CCGTCGTGTAGTCTGTCTCGTA |  |  |
| *NF-κB* | Forward | CCTTCCGCAAACTCAGCTTT | 173 | NM_008689.2 |
|  | Reverse | GGACGATGCAATGGACTGTC |  |  |
|  | Reverse | CCGTCGTGTAGTCTGTCTCGTA |  |  |

**Results**

**Table S2 MIC of CNMs against pathogenic bacteria**

| **Strains** | **MIC (%)** |
| --- | --- |
| **Tet-resistant ETEC CVCC1522** | **0.025** |
| ***E. coli* K88** | **0.05** |
| ***Salmonella pullorum* CVCC1791** | **0.05** |
| ***Salmonella typhimurium* ATCC14028** | **0.05** |
| ***Salmonella enteritidis* CVCC3379** | **0.10** |

| **Items** | **DPPH（IC50, µg/mL)** |
| --- | --- |
| **VC** | **13.58** |
| **BHT** | **38.62** |
| **CNMs** | **418.75** |

**Table S3 DPPH free radical half clearance of CNMs**
